# Organizational characteristics of nursing practice environments related to registered nurses’ professional autonomy and job satisfaction in two Finnish Magnet-aspiring hospitals: structural equation modeling study

**DOI:** 10.1186/s12912-024-01772-9

**Published:** 2024-02-06

**Authors:** Katja Pursio, Päivi Kankkunen, Santtu Mikkonen, Tarja Kvist

**Affiliations:** 1 Department of Nursing Science, Faculty of Health Sciences, University of Eastern Finland, Kuopio, Finland; 2 Department of Applied Physics, and Department of Environmental and Biological Sciences, University of Eastern Finland, Kuopio, Finland

**Keywords:** Instrument validation, Job satisfaction, Magnet-aspiring hospital, Nursing practice environment, Nursing Work Index-Revised, Professional autonomy, Registered nurse, Structural equation modeling

## Abstract

**Background:**

Nurses are leaving their profession because of poor personal job satisfaction, heavy workload, and unfavorable work environments with low professional autonomy. Professional autonomy involves the possibility to influence one’s work and have a sense of control – the ability to contribute to a workplace culture and influence how decisions are made. This study explores registered nurses’ perceptions of the nursing practice environment, using the Nursing Work Index-Revised (NWI-R), and its relationships with professional autonomy and job satisfaction.

**Methods:**

A cross-sectional study along with instrument re-validation was conducted using a web-based survey for nurses in two Magnet-aspiring hospitals in Finland in September 2021 (*n* = 586). Structural equation modeling was used to find out the relationships of the NWI-R components with professional autonomy and job satisfaction.

**Results:**

Principal component analysis and confirmatory factor analysis supported seven components with 34 items. Collegial nurse–doctor relationships, organization’s quality standards, and nursing involvement and expertise sharing (means of 3.23, 2.96, and 2.66, respectively) demonstrated a favorable nursing practice environment; professional nursing standards, nurse management and leadership, staffing and resource adequacy, and professional advancement (means of 2.38, 2.18, 2.15, and 2.13, respectively) demonstrated an unfavorable nursing practice environment. The presented model (RMSEA 0.068, CFI 0.987, TLI 0.946) indicated that nursing involvement and expertise sharing, organization’s quality standards, nurse management and leadership, and collegial nurse–doctor relationships were related to professional autonomy. Nurse management and leadership, staffing and resource adequacy, and organization’s quality standards were related to job satisfaction. Moreover, professional autonomy was related to job satisfaction.

**Conclusion:**

Nurses’ professional autonomy is important due to its relationship with job satisfaction. When factors that increase professional autonomy are taken into account and attention is paid to the promotion of autonomy, it is possible to improve nurses’ job satisfaction. These issues cannot be solved at the unit level; investment is needed at the organizational and political levels. The results introduce nurses, managers, researchers, and stakeholders to improvements in the nursing practice environment toward an organizational culture where nurses may utilize their professional autonomy to its full potential.

**Supplementary Information:**

The online version contains supplementary material available at 10.1186/s12912-024-01772-9.

## Background

There is already a huge shortage of nurses globally, and the number of graduating nurses is not enough to compensate for the number of retiring nurses, leading stakeholders to search for inducements for nurses to work beyond the retirement age [1]. Nurses are leaving the profession due to poor personal job satisfaction, heavy workloads, and a lack of adequate resources. Younger nurses in particular have reported lower levels of organizational commitment and higher levels of intention to leave than their older colleagues [2]. Research should examine nurses’ perceptions of opportunities to influence their own work—professional autonomy—and experiences of job satisfaction.

Professional autonomy is notable for nurses who develop their activities and experience, face challenges, and co-participate in decision making. Professionally autonomous nurses provide high-quality patient care while taking care of themselves [3]. The experience of professional autonomy includes the possibility to influence one’s work and have a sense of control; it is part of nurses’ intrinsic work motivation. The possibility of having influence over work refers to the ability to contribute to workplace culture and influence processes and decision making [4]. A sense of control refers to certainty about the continuation of work. Thus, professional autonomy means the right to self-determination, which is an individual factor affecting job satisfaction and organizational factors like management or resources [5]. Nurses’ professional autonomy is reduced by lack of resources, reliance on other services, segregation between nurses and doctors, and lack of institutional support [6].

There have been shortages of nurses throughout history, especially during the 1980s, when the so-called Magnet ideology was prominent, and some hospitals had noticeably less trouble attracting and retaining talented and committed nurses. The Magnet framework fostered high levels of job satisfaction, respect and autonomy in practice, professional governance, and appropriate resources. Four decades later, it is still an efficient model for nurse retention, providing tools for responding to challenges and creating positive organizational cultures [4, 7]. The Magnet Recognition Program model, presented by the American Nurses Credentialing Center (ANCC), contains five components configured with 14 forces of magnetism: transformational leadership, structural empowerment, exemplary professional practice, new knowledge, innovation and improvements, and empirical quality results. Autonomy is included in the exemplary professional practice component, and nurses’ job satisfaction is part of empirical quality results [8]. Magnet culture supports investment in nursing education, development, and chosen career paths. To nurses, this means progress through every career stage, which leads to greater autonomy in their practice. Interprofessional collaborative practice is appreciated, with a focus on relative respect, autonomy, and shared values. In Magnet hospitals, job satisfaction among nurses has been found to be higher [4, 9].

Interest in the Magnet model has influenced nursing management in Finnish health care, and the same ideology is included in nursing strategies in Finland [1]. Kanninen et al. [10] found that nurse managers felt that decision-making power in daily nursing matters was extended to the nurses, and they have good control over their practice, as well as the ability to set goals and resolve issues. However, in the same study, nurses reported that they hardly have any impact in decision making and that their organization’s governance style was traditional. A discrepancy exists between the views of the nurses and those of their managers regarding the reality of the nursing practice environment and nurses’ opportunities to influence health care in Finland. Several Magnet hospital studies have explored how favorable or unfavorable nursing practice environments are for nurses. Instruments such as the Nursing Work Index-Revised (NWI-R) and the Practice Environment Scale (PES) have been widely used to describe the organizational characteristics of a nursing practice environment. Aiken and Patrician [11] and Lake [12] are pioneers in this area, and their instruments have been modified numerous times. This study explores nursing practice environments using the NWI-R because its subscale scores (including autonomy) have been associated with, among other things, nurses’ job satisfaction and high levels of retention [13].

### Aims

The study aims to explore registered nurses’ perceptions of nursing practice environments using the re-validated Nursing Work Index-Revised (NWI-R), examining its relationships with professional autonomy and job satisfaction in two Magnet-aspiring university hospitals in Finland. The study addresses the following research questions:

1. How valid is the structure of the NWI-R in a Finnish context?
2. How are nursing practice environments, professional autonomy, and job satisfaction perceived by registered nurses?
3. Which organizational characteristics of a nursing practice environment are related to nurses’ professional autonomy and job satisfaction?

## Methods

### Design

The study used a descriptive cross-sectional design along with instrument re-validation. The study was reported following the STROBE checklist.

### Participants and data collection

Two university hospitals in Finland are Magnet-aspiring hospitals. At Hospital A, four large departments (Children and Adolescents, Comprehensive Cancer Center, Heart and Lung Center, and Psychiatry) are in the Magnet Recognition Program. Hospital B aspires for Magnet status as a whole organization. A total of 4,400 registered nurses were invited to participate in the survey from four departments of Hospital A and across the entirety of Hospital B—2,730 and 1,700 nurses, respectively. Contact persons sent an electronic survey with an introduction to the participants in September 2021. The minimum number of responses required was calculated using power analysis with a 95% confidence interval (*n* = 354) and achieved with a sample size of 586 (13.3% response rate).

### Instruments

A self-administered questionnaire consisted of three sections: a demographic data section and two translated and validated international instruments. The demographic data portion included age, gender, education, years of nursing experience, years in current unit, organization, unit type, and day/shift work. Job satisfaction was assessed regarding their current experience, on a scale of 0–10.

The Dempster Practice Behavior Scale (DPBS) explores the full expression of nurses’ professional autonomy. This instrument was developed to address the lack of useful and generalizable tools for understanding, prediction, and control related to autonomy in practice [14]. This study used the previously translated and validated Finnish version of the DPBS (FI-DPBS). It includes 24 items out of the original 30 items in Dempster’s instrument. The FI-DPBS describes the existence of professional autonomy through five dimensions: actualization, valuation, authority, empowerment, and readiness. The instrument has a five-point Likert-like scale from 1 (not at all true) to 5 (extremely true), from which the respondents chose the best option that indicates their practice. The scale content validity index, CVI/average, of the FI-DPBS has been estimated as 0.94, and the reliability analysis indicates a Cronbach’s α value of 0.89. The validation details of the FI-DPBS and survey results have been presented elsewhere; here, we report only the total professional autonomy score, which has been examined previously.

The NWI-R was used to measure the supportive organizational characteristics of professional nursing practice environments. The Nursing Work Index was devised in the mid-1980s to assess work environment issues related to nurses’ job satisfaction and quality of care based on the findings of Magnet hospitals. The focus on the organization’s environmental factors led to the development of the NWI-R. [11] It consists of four subscales (autonomy, control over practice settings, nurse–physician relationships, and organizational structures) with 57 items. In Finland, the NWI-R (55 items out of 57) has been previously translated and used—for example, by Tervo-Heikkinen et al. [15] and Hinno et al. [16]. However, these studies were conducted over a decade ago, and the nursing practice environment has undergone significant changes, so re-validation was necessary. We added one question from the original NWI-R to the Finnish translated version; thus, the total number of NWI-R items was 56 in our study. The NWI-R has a four-point Likert-like scale from 1 (strongly agree) to 4 (strongly disagree). The respondents were asked to assess the presence of each item. The original NWI-R demonstrated good internal consistency reliability with a Cronbach’s α of 0.96 [11]. Despite cultural differences and various factor structures in previous studies (see Supplementary file 1), the NWI-R remains a highly validated and extensively used instrument to explore nurses’ practice environments internationally [16].

### Data analysis

The demographic data were analyzed using descriptive statistics (frequency, percentage, mean, standard deviation, and minimum–maximum values). The total professional autonomy and job satisfaction were analyzed using the mean scores of all the FI-DPBS items and experienced job satisfaction, respectively.

Principal component analysis (PCA) as a data dimension reduction method was used to identify the structure of the NWI-R (i.e., the organizational characteristics of nursing practice environments). As in previous NWI-R studies, all the items were first reverse-coded so that higher numbers indicated stronger agreement. Missing single values (*N* = 34) were observed. The varimax method was used as the rotation technique. The number of components was decided based on the number of eigenvalues greater than one, the scree plot, and the cumulative percentage of variance explained by the extracted components and component loading values of the rotated component matrix. An item’s inclusion in a component was based on communality and a component loading of 0.30 or more [17].

The mean scores for each component were calculated. To interpret the NWI-R component scores, 2.50 was considered a neutral value on the four-point response scale, scores lower than 2.50 signified unfavorable nursing practice environments, and scores higher than 2.50 indicated favorable nursing practice environments [12]. Internal consistency reliability was examined by computing Cronbach’s alpha values for each component and the entire scale. Reliability coefficients more than 0.80 are considered strong, while 0.60 is considered low and as indicating limited instrument reliability [18].

**Fig. 1:**
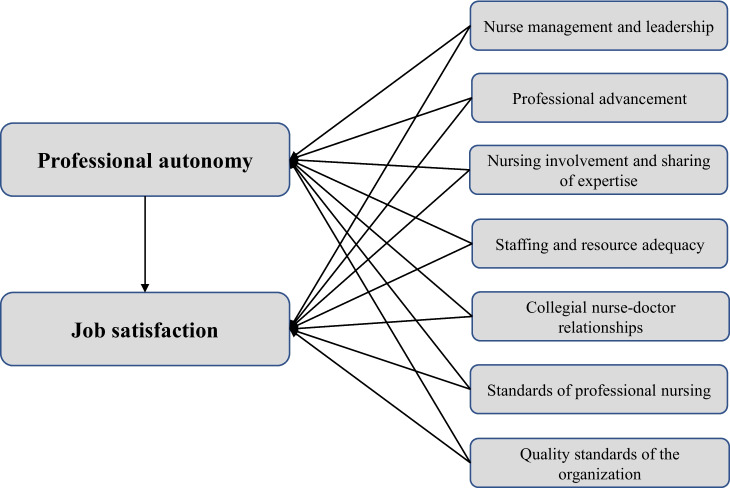
Hypothesized model

Then, a confirmatory factor analysis (CFA) with maximum likelihood extraction was conducted to test and confirm the fit of the data to the model obtained through the PCA. Structural equation modeling (SEM) was used to test the hypothesized model (Fig. 1) that NWI-R components would have effects on nurses’ professional autonomy and job satisfaction. SEM involved five model testing steps: model specification, identification, estimation, evaluation, and modification [19]. Maximum likelihood extraction was chosen. Based on the statistical significance of parameter estimates and an examination of modification indices (MI), the paths were deleted from or added to the model one by one. Total, direct, and indirect effects were computed. Direct effect means a connection between two variables, which is not necessarily causal; indirect effect means a connection through some other variable. To assess the model adequacy in both CFA and SEM, the root mean square error of approximation (RMSEA), the comparative fit index (CFI), and the Tucker–Lewis fit index (TLI) were used. Acceptable model fit was considered for RMSEA values < 0.1 and for CFI and TLI values > 0.9 [20]. The statistical analyses were performed together in SPSS Statistics 27 for Windows and SPSS AMOS version 27.0 (SPSS Inc., Chicago, IL).

### Ethical considerations

The Committee on Research Ethics of the authors’ academic institution approved the study in March 2021, and both organizations granted research permissions in May 2021. The copyright holder of the original instruments granted permission to use them in data collection in December 2020. Also, permission to use the Finnish version of the NWI-R was ensured from the particular university. Data collection was performed anonymously using an electronic link with the help of contact persons. Informed consent was obtained from all the participants.

## Results

### Demographic characteristics

The sample consisted of 586 registered nurses from two Finnish university hospitals. Most of the respondents were female (85.7%), with a mean age of 42.3 years (SD 11, a range of 22–65). Regarding education, 67.4% of the nurses had the highest degree from a university of applied sciences. The mean work experience as a nurse was 15.1 years (SD 10.9). The participating nurses worked in an outpatient department (30.0%); emergency room (5.6%); intensive care unit (13.3%); operating room, day surgery, or day hospital (9.2%); or hospital ward (35.3%). Their work experience in their current unit was an average of eight years (SD 7.9). Among the participants, 62.1% worked shifts. The participants’ demographic descriptions are summarized in Table 1.

**Table 1:** Nurses’ demographic descriptions (*N* = 586, n, %, mean, SD)

Participants’ demographic descriptions	n (%)	Mean (SD)
Gender		
Female	502 (85.7)	
Male	73 (12.4)	
Other or undisclosed	11 (1.9)	
Age		42.3 (11.0)
Highest nursing degree		
Registered nurse	68 (11.6)	
Registered nurse with specialization	77 (13.1)	
University of applied sciences	395 (67.4)	
Master’s degree (MSc) or PhD	46 (7.9)	
Organization		
Hospital A	360 (61.4)	
Hospital B	226 (38.6)	
Unit type		
Outpatient department	176 (30.0)	
Emergency room	33 (5.6)	
Intensive care unit	78 (13.3)	
Operation room, day surgery, or day hospital	54 (9.2)	
Hospital ward	207 (35.3)	
Other	38 (6.6)	
Years of nursing experience		15.1 (10.9)
Years in current unit		8.0 (7.9)
Shifts		
Only day shifts	222 (37.9)	
All shifts	364 (62.1)	

### Re-validation of the Nursing Work Index-Revised (NWI-R)

Two variables did not correlate with any of the other variables (*r* > 0.30); thus, PCA was conducted for the NWI-R with 54 items. The result contained seven components with 34 items; each component had a coherent set of 4–7 items (Table 2). The Kaiser–Meyer–Olkin test value was 0.92, and Bartlett’s test of sphericity was statistically significant (*p* < 0.001). All the components demonstrated eigenvalues greater than one (1.1–10.4), and they explained 60.4% of the total variance (4.4–9.8). All seven components were relevant in describing the organizational characteristics of nursing practice environments, and they were named as follows: nurse management and leadership, professional advancement, nursing involvement and expertise sharing, staffing and resource adequacy, collegial nurse–doctor relationships, professional nursing standards, and the organization’s quality standards.

**Table 2:** Components, scores, and Cronbach’s α of the Nursing Work Index-Revised (NWI-R) (*n* = 586)

Components and items	Loading^a^	Mean score^b^ (SD)	Cronbach’s alpha
**Nurse management and leadership**		**2.18 (0.66)**	**0.86**
A nurse manager who is a good manager and leader	0.816	2.63 (0.97)	
Nurse managers consult with the staff on daily problems and procedures	0.781	2.66 (0.97)	
A nurse manager backs up the nursing staff in decision making, even if the	0.761	2.56 (0.92)	
conflict is with the doctor			
The managerial staff are supportive of the nurses	0.522	1.99 (0.90)	
Praise and recognition for a job well done	0.441	2.19 (0.84)	
A chief nurse executive is highly visible and accessible to the staff	0.330	1.67 (0.86)	
An administration that listens and responds to the employee’s concerns	0.356	1.53 (0.75)	
**Professional advancement**		**2.13 (0.76)**	**0.87**
Career development ladder opportunity	0.852	2.00 (0.91)	
Opportunity for advancement	0.821	2.10 (0.91)	
Active in-service/continuing education program for nurses	0.706	2.16 (0.86)	
The nursing staff are supported in pursuing degrees in nursing	0.638	2.27 (0.88)	
**Nursing involvement and expertise sharing**		**2.66 (0.55)**	**0.77**
Freedom to make important patient care and work decisions	0.685	2.74 (0.72)	
The staff nurses are involved in the internal governance of the hospital	0.650	2.36 (0.83)	
(e.g., practice and policy committees)			
The staff nurses have the opportunity to serve on hospital and nursing	0.637	2.91 (0.75)	
committees			
Opportunity for the staff nurses to participate in policy decisions	0.575	2.61 (0.85)	
The nursing staff participate in selecting new equipment	0.490	2.32 (0.86)	
Support for new and innovative ideas about patient care	0.459	3.01 (0.82)	
**Staffing and resource adequacy**		**2.15 (0.72)**	**0.80**
Enough registered nurses or staff to provide quality patient care	0.847	1.87 (0.89)	
Enough staff to get work done	0.797	1.97 (0.94)	
Adequate support services that allow me to spend time with my patients	0.643	2.24 (0.91)	
Enough time and opportunities to discuss patient care problems with other	0.632	2.51 (0.89)	
nurses			
**Collegial nurse–doctor relationships**		**3.23 (0.53)**	**0.78**
Collaboration (joint practice) between nurses and doctors	0.810	3.21 (0.69)	
Much teamwork between doctors and nurses	0.795	3.18 (0.76)	
The doctors and nurses have a good working relationship	0.767	3.12 (0.74)	
The doctors give high-quality medical care	0.494	3.28 (0.72)	
The nurses control their own practices	0.308	3.34 (0.71)	
**Professional nursing standards**		**2.38 (0.64)**	**0.63**
Use of nursing diagnosis	0.778	2.29 (1.03)	
Nursing care is based on a nursing rather than medical model	0.626	2.55 (0.77)	
Written, up-to-date nursing care plans for all patients	0.534	2.47 (0.92)	
Clinical nurse specialists who provide patient care consultations	0.460	2.20 (0.94)	
**Organization’s quality standards**		**2.96 (0.55)**	**0.59**
Opportunity to work on a highly specialized unit	0.642	3.28 (0.78)	
Standardized policies, procedures, and ways of doing things	0.566	2.73 (0.75)	
Not having to do things that are against my nursing judgment	0.358	2.76 (0.82)	
High standards of nursing care expected by the administration	0.465	3.05 (0.92)	
Total Cronbach’s α for 34 items			**0.93**

^a^Principal component analysis, Varimax with Kaiser normalization, explained 60.4% of the total variance

^b^ Scale: 1 = lowest, 4 = highest

The seven components of the NWI-R found in the PCA were subjected to CFA. To improve the model, some error correlations between two items were introduced based on the largest values of modification indices (MI). Thus, the CFA presented a model with a good fit to the data (RMSEA 0.039, CFI 0.947, TLI 0.935).

Internal consistency reliability was strong in the components of professional advancement (0.87), nurse management and leadership (0.86), and staffing and resource adequacy (0.80). It was moderate in collegial nurse–doctor relationships (0.78), nursing involvement and expertise sharing (0.77), and professional nursing standards (0.63). Internal consistency reliability was slightly low (0.59) in organization’s quality standards. Cronbach’s alpha for the entire scale was 0.93. Thus, based on these findings the structure of the NWI-R is valid in a study context.

### Organizational characteristics of professional nursing practice environments

The mean scores of the components varied from 2.13 to 3.23 out of 4 (Table 2). According to cut-off point as 2.5 [12] three components demonstrated favorable nursing practice environments: collegial nurse–doctor relationships, 3.23 (SD 0.53); organization’s quality standards, 2.96 (SD 0.55); nursing involvement and expertise sharing, 2.66 (SD 0.55). Instead, four components demonstrated unfavorable nursing practice environments: professional nursing standards, 2.38 (SD 0.64); nurse management and leadership, 2.18 (SD 0.66); staffing and resource adequacy, 2.15 (SD 0.72); and professional advancement, 2.13 (SD 0.76).

### Professional autonomy and job satisfaction

According to the FI-DPBS, the professional autonomy of the nurses was 3.6 (SD 0.5) on a scale of 1–5. The autonomy score can be interpreted as above mid-range, even though there are not defined low, moderate, or high levels for the scale. The findings on the dimensions of professional autonomy and the relationship of the background variables to them have been presented elsewhere. The average evaluation of job satisfaction among the nurses was 6.6 (SD 2.1) on a self-assessment scale of 0–10.

### Organizational characteristics of nursing practice environments related to nurses’ professional autonomy and job satisfaction

The improved model demonstrated that *nurse management and leadership, nursing involvement and expertise sharing, collegial nurse–doctor relationships, and organization’s quality standards* have positive relationships with nurses’ professional autonomy. It also confirmed that *nurse management and leadership, staffing and resource adequacy, and organization’s quality standards* have positive relationships with nurses’ job satisfaction. Moreover, professional autonomy has a strong connection to job satisfaction.

**Fig. 2:**
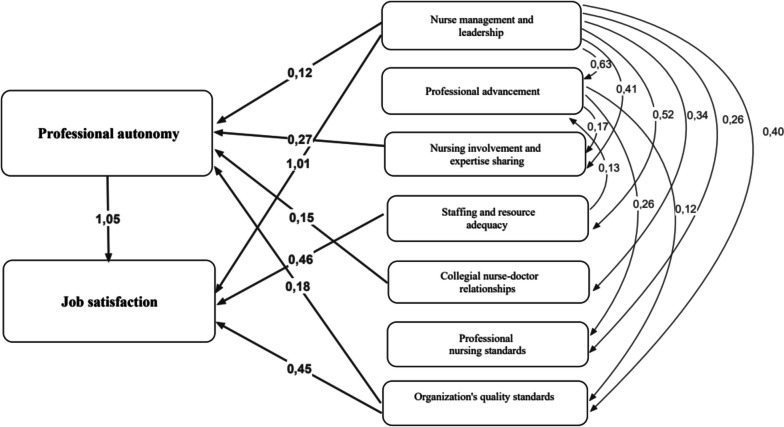
Organizational characteristics of professional nursing practice environments related to nurses’ professional autonomy and job satisfaction (direct effects)

*Professional advancement* and *professional nursing standards* had no statistically significant relationships with either professional autonomy or job satisfaction, but professional advancement was indirectly related to both. Professional nursing standards was, however, directly and indirectly related to other independent variables (nurse management and leadership, staffing and resource adequacy, professional advancement), which is why it was left in the model. The presented model fits the data well (RMSEA 0.068, CFI 0.987, TLI 0.946). Table 3 shows the standardized total, direct, and indirect effects, and Fig. 2 presents the direct effects. Regression weights, both unstandardized and standardized, can be found in Supplementary file 2.

**Table 3:** Standardized total, direct, and indirect effects of organizational characteristics of nursing practice environments on nurses’ professional autonomy and job satisfaction

Path	Standardized Total Effects	Standardized Direct Effects	Standardized Indirect Effects
Professional autonomy			
Nurse management and leadership	0.544	0.165	0.379
Professional advancement	0.102	0	0.102
Nursing involvement and expertise sharing	0.301	0.301	0
Staffing and resource adequacy	0.013	0	0.013
Collegial nurse-doctor relationships	0.166	0.166	0
Organization’s quality standards	0.206	0.206	0
Job satisfaction			
Nurse management and leadership	0.586	0.314	0.272
Professional advancement	0.043	0	0.043
Nursing involvement and expertise sharing	0.073	0	0.073
Staffing and resource adequacy	0.162	0.157	0.005
Collegial nurse-doctor relationships	0.040	0	0.040
Organization’s quality standards	0.167	0.117	0.050
Professional autonomy	0.242	0.242	0

RMSEA 0.068, CFI 0.987, TLI 0.946, *p* < 0.01 or *p* < 0.001

## Discussion

### The structure of the NWI-R

The NWI-R focuses on an organization’s environmental factors, and it has been used widely in a variety of countries. However, it has also been criticized for its factor structure [12, 13]. Researchers have argued that the NWI-R and the original set of theoretically derived four subscales including 15 items may be poorly generalizable to new settings, which has led to various factor structures in previous studies, especially in the early 2000s. Since the nursing practice environment is culturally bound and has generally changed significantly in recent years (e.g., shortage of nurses, leadership, the spread of the Magnet hospital model, the new generation of nurses and their expectations), we justify its re-validation. Despite the criticisms, our choice to use the NWI-R in this study is bolstered by the fact that one of its original subscales was autonomy. In later factor structure definitions by other researchers, the nursing management subscale was often formed from the items included in the autonomy subscale, and it ultimately replaced autonomy [13]. To get an overall picture of a complex and multifaceted nursing practice environment, we also performed PCA, which best explained the components for particular data. Instead of forcing items to a certain number of components based on previous research, we allowed items to load freely for components considering the moderately low loadings (> 0.3), initially allowing cross-loadings as well. Our result of the seven-component structure with 34 items provided a comprehensive but condensed profile of key dimensions in the nursing practice environment in Finland. The CFA confirmed the structure given by the PCA. Autonomy did not form its own component in our NWI-R analysis; the items were evenly placed into the four components of nurse management and leadership, collegial nurse–doctor relationships, nursing involvement and expertise sharing, and organization’s quality standards. Items under these components describe how much control nurses have over practice, how nurse managers support that control, and how relationships with doctors permit or hinder that control [13]. Remarkably, all four components were related to nurses’ professional autonomy in this study according to structural equation modeling.

Internal consistency reliability assessed by calculating Cronbach’s α values for components varied from 0.59 to 0.87. The value of Cronbach’s α in organization’s quality standards was slightly less than 0.6, which is one threshold for low reliability [18]. We accepted it nonetheless, including four items. Low Cronbach’s α could be due to a low number of items, poor correlations between items, or heterogeneous constructs [21]. Even if the standard deviation (0.55) of the component mean score seems to be reasonable and does not differ from other components, the nurses may have had different views on the items, which would explain the low Cronbach’s α. The overall Cronbach’s α for the NWI-R was high (0.93), which shows that the instrument was internally reliable. In summary, the psychometric testing verified that the NWI-R is a valid and reliable instrument to measure the presence of organizational characteristics of nursing practice environments in study hospitals.

### Organizational characteristics of nursing practice environments

NWI-R components refer to organizational characteristics of nursing practice environments. For reporting and analyzing the results of the organizational characteristics of nursing practice environments, we used 2.5 as the neutral value or cut-off point for defining the practice environment as unfavorable or favorable [12]. In our study, three components—namely, collegial nurse–doctor relationships (3.23), organization’s quality standards (2.96), and nursing involvement and expertise sharing (2.69)—were above the neutral value and described a favorable environment. However, only collegial nurse–doctor relationships achieved a value of 3, which corresponded with agreement in the response options. Hence, nursing involvement and expertise sharing, and organization’s quality standards suggested low to moderate agreement with the items of particular components [12]. Similar results on the clear positive nurse–doctor relationship have been found in several studies [e.g., [4, 17]. The power of the nurses’ professional autonomy arises from the basis of self-knowledge that is not a subordinate of medical practice [6]. In Western countries, nurses generally have good authority. These three favorable dimensions of the nursing practice environment form a whole. Sharing one’s own expertise and working in collaboration with doctors in an organization with high quality standards establish favorable working conditions for nurses. Improving work environments and achieving optimal multi-professional teamwork are also important parts of the Magnet journey to create a culture of excellence [4, 8].

Nurses disagreed with the NWI-R component items included in professional advancement (2.13), staffing and resource adequacy (2.15), nurse management and leadership (2.18), and professional nursing standards (2.38). In these respects, the nursing practice environment can be considered as unfavorable. This finding was expected, in some ways. The extensive shortage of nurses has led to heavy workloads and a lack of adequate resources [2], and a traditional governance style is still apparent in many ways [10]. Even if the nurses in this study assessed nurse management and leadership, professional advancement, staffing and resource adequacy, and professional nursing standards to be at lower levels, they still experienced the organization’s quality standards as being high. Nonetheless, future improvements following the results of Magnet hospitals are necessary for all aspects of the nursing practice environment, emphasizing shared governance and the assurance of adequate resources [4, 7]. However, understanding and implementing the principles of shared governance necessitates training for both nurses and managers. Interestingly, Kanninen et al. [10] reported that nurse leaders and experts already have rather strong perceptions about staff input in the governance of health care organizations, but other groups have not noticed the change yet. Our results confirm that the involvement of clinical nurses needs to be clarified.

### The relationship between organizational characteristics of nursing practice environments with nurses’ professional autonomy and job satisfaction

This study’s main interest was to determine which organizational characteristics are related to nurses’ professional autonomy and job satisfaction. According to structural equation modeling, nurse management and leadership, nursing involvement and expertise sharing, collegial nurse–doctor relationships, and organization’s quality standards had statistically significant relationships with nurses’ professional autonomy. These findings, which highlight nursing involvement and expertise sharing expertise as the strongest, suggest that involving nurses and giving them opportunities to influence can increase autonomy even more. In recent years, Magnet-aspiring hospitals in Finland have developed practice and policy committee activities, providing staff nurses with opportunities to participate in them. At the same time, the involvement of nurses in the development of their own work has been systematized. It can be deduced that this development is reflected in our results, and registered nurses recognize their potential for influence. This is an important finding, since nurses feel that they have a voice in their practice in work units where shared governance and nursing empowerment are strengthened. Moreover, regarding Magnet components, structural empowerment requires evidence pointing at structures that include registered nurses in development teams, councils, and committees at different levels of the organization [7].

In our study, the variable of nurse management and leadership also had relationships with all other independent variables—i.e., the organizational characteristics of nursing practice environments. The strongest connections were to professional advancement and to staffing and resource adequacy. Hence, management (including the leadership style, culture, and individual characteristics of leaders) is definitely relevant to professional autonomy. A recent study of Finnish nurse managers’ work reported similarities within the Magnet model structure—such as transformational leadership, shared governance, and structural empowerment [1]—that support our result on the connection between nurse management and professional autonomy. What is notable, however, is that nurses disagreed with the component of nurse management and leadership (2.18). Improvements in transformational leadership, shared governance, and structural empowerment are essential to achieving better professional autonomy, but work being managed from above clearly reduces the sense of autonomy.

Nurses in this study assessed their job satisfaction with a mean score of 6.6, which cannot be considered satisfactory. However, the result is based on only one numerical question, which makes us unable to analyze what job satisfaction consists of and to discuss the factors that contributed to the relatively low result. Nurse management and leadership has a strong statistically significant relationship with job satisfaction. This finding is similar to the results from previous studies [e.g. [22, 23]. Leadership style at all levels of an organization is a major consideration for increasing job satisfaction. It is important for nurses to feel that they are valued professionals instead of being pressured, monitored, and ignored. In this study, staffing and resource adequacy, and quality standards of the organization were also related to job satisfaction. Accordingly, nurses appreciate working with their full professional potential in a high-quality organization, giving enough time to caring for their patients. The low job satisfaction result might also be explained by the shortage of nurses and by challenges in staffing and resource adequacy.

Collegial nurse–doctor relationships did not have a connection to job satisfaction. This NWI-R component nevertheless received the highest mean scores and demonstrated a favorable nursing practice environment; thus, this was a rather surprising result. For example, Galletta et al. [24] found that nurse–doctor collaboration is positively related to nurses’ job satisfaction and negatively related to turnover intention in Italy. Moreover, similar results have also been found earlier in South-Korea and Brazil [25, 26]. Schmalenberg and Kramer [27] revealed that collegial and collaborative nurse–doctor relationships were more common in Magnet hospitals than in non-Magnet hospitals, and that high-quality nurse-doctor relationships increased job satisfaction among nurses in addition to patient outcomes and nurses’ autonomy. In Finland, turnover among doctors is quite high; also, given their educational paths, there might be turbulence in multi-profession teams. In other words, nurses work with several doctors in varying periods. Other characteristics related to the work environment might be highlighted with job satisfaction instead of nurse–doctor relationships.

Another surprising result was that professional advancement had no statistically significant connection to either nurses’ professional autonomy or job satisfaction and were only indirectly connected to nursing involvement and expertise sharing, professional nursing standards, and organization’s quality standards. Still, professional advancement by offering professional development programs, for example, is important to enhancing nurses’ autonomy and increasing occupational commitment [3]. Professional advancement through further education, certifications, and continued training is also included in the Magnet journey [4]. Recent findings have demonstrated higher proportions of certified and continuing educated nurses, especially in Magnet hospitals [28, 29]. Thus, it is unfortunate that nurses in this study disagreed with NWI-R items related to the component of professional advancement (2.13); the associations to professional autonomy or job satisfaction might not have been clearly presented. However, if the offered update courses and continuing education program only meet the organization’s interests, this does not promote nurses’ professional autonomy [6] or job satisfaction. Organizations often recommend and require training programs for nurses, and some of them might be prerequisites for work. Dealing with this in situations where resources are scarce is important, as it may be impossible to find time to educate themselves; nurses might miss out on many useful and motivating voluntary courses. This leads back to basic issues such as supportive management, adequate resources, and asking the nurses about which programs they personally find to be necessary and motivating.

Finally, the association found between nurses’ professional autonomy and job satisfaction strengthens the findings of previous studies [3, 30]. It is worth noting that this association was the strongest positive relationship in the whole model. When factors that increase professional autonomy are taken into account and attention is paid to the promotion of autonomy, it is possible to improve nurses’ job satisfaction.

Findings of favorable nursing practice environments in Magnet hospitals are undoubtedly extensive, especially in the United States. They guide us in the Magnet journey and make recommendations for developing an excellent organizational culture for nurses where their professional roles and appropriate autonomy can flourish. However, more studies are needed outside of the United States. In some respects, there are challenges to implementing North American ideology and structures related to Magnet hospitals in international health contexts because of educational mandates, clinical practices, staffing levels, and cost models [4]. Our study presented the status of the nursing practice environment and its relationship to nurses’ professional autonomy and job satisfaction in two Magnet-aspiring university hospitals in Finland. More research is needed on both nursing practice environments and professional autonomy, along with a longitudinal study of their connections and impact on job satisfaction.

### Limitations

Our study has some limitations. First, given our focus on Magnet-aspiring hospitals, the participating nurses were only from two organizations. Noting that the NWI-R assesses organizational phenomena surrounding nurses from both hospital-level and unit-level perspectives, some components and their items deal with the hospital level, which limited the re-evaluation of the instrument. Thus, the results should be considered as indicative only and should not be generalized. Second, despite a sufficient number of participants, the response rate was low (13.3%). After over two years of the COVID-19 pandemic and a hectic work pace with few resources in hospitals, nurses’ opportunities and eagerness to respond to simultaneous surveys vary. The low response rate may introduce a sociodemographic selection bias. Third, self-assessment may cause underestimation or overestimation biases. Fourth, the components of the NWI-R are based on the PCA of this particular data presenting organizational characteristics of the nursing practice environment. The NWI-R has numerous factor structures internationally; although the concepts are often similar, the scores cannot be directly compared. Finally, structural equation modeling offers a summary of the interrelationships among variables, but the result is always context-dependent and affected by deleting or adding predictors. In addition, causality cannot be determined due to the cross-sectional design.

## Conclusion

Many factors must be considered to achieve a favorable environment for nursing. The results presented highlight that professional autonomy is clearly related to job satisfaction, which is why promoting nurses’ autonomy is important. Issues related to management, nursing involvement, nurse–doctor relationships, and organization’s quality standards should be carefully considered to improve nurses’ professional autonomy. While development according to the Magnet hospital framework has begun to produce results, and nurses recognize their potential for influence, more effort is needed to involve nurses in decision making and bring their expertise to the fore regarding care and working conditions. This must be done in close cooperation with doctors, nursing leaders, and hospital management.

The results also highlight room for improvement in job satisfaction. In addition to paying attention to strengthening professional autonomy, it is essential to ensure empowering and encouraging management and leadership, adequate resources, and high organization quality standards, which are all related to nurses’ job satisfaction. These issues cannot be solved only at the unit level; an investment is needed at the organizational and political levels as well. The results will introduce nurses, nurse managers, researchers, and stakeholders to improvements in the nursing practice environment toward an excellent organizational culture where nurses may utilize their professional autonomy to its full potential and experience job satisfaction at the same time.

### Supplementary Information

**Additional file 1.**

**Additional file 2.**

## Data Availability

The data is not publicly available due to privacy or ethical restrictions. The data sets used and analyzed are available from the corresponding author on reasonable request.

## Abbreviations

ANCC: American Nurses Credentialing Center
CFA: Confirmatory Factor Analysis
CFI: Comparative Fit Index
COVID-19: Coronavirus Disease 2019
CVI: Content Validity Index
DPBS: Dempster Practice Behavior Scale
FI-DPBS: Finnish version of the Dempster Practice Behavior Scale
MI: Modification Indices
NWI-R: Nursing Work Index-Revised
PCA: Principal Component Analysis
PES: Practice Environment Scale
R: Correlation
RMSEA: Root Mean Square Error of Approximation
SD: Standard Deviation
SEM: Structural Equation Modeling
STROBE: Strengthening the Reporting of Observation studies in Epidemiology
TLI: Tucker–Lewis Fit Index
