# Supplementary material for: Organizational characteristics of nursing practice environments related to registered nurses’ professional autonomy and job satisfaction in two Finnish Magnet-aspiring hospitals: structural equation modeling study
Source: BMC Nurs. 2024 Feb 6;23:100. doi: 10.1186/s12912-024-01772-9 (PMC10845793; doi:10.1186/s12912-024-01772-9)
Supplement: Supplementary file 1 — Additional file 1. [file 12912_2024_1772_MOESM1_ESM.docx]

Supplementary file 1. Previous findings from Nursing Work Index-Revised subscales and items.

| Study | Factors | Subscales (Factor names) | Items |
| --- | --- | --- | --- |
| Aiken & Patrician (2000), USA | 4 | Autonomy, nurse-doctor relationship, control over practice, organizational support | 15 |
| Estabrooks et al. (2002), Canada | 1 | Practice environment | 26 |
| Lake (2002), USA | 5 | Staffing and resources adequacy, collegial nurse-doctor relations, nurse manager ability/leadership/support of nurses, nurse participation in hospital affairs, nurse foundations for quality of care | 30 |
| Choi et al. (2004), USA | 7 | Staffing and resources adequacy, nurse-doctor relationship, nurse management, professional practice, nursing competence, nursing process, positive scheduling climate | Unclear |
| McCusker et al. (2004), Canada | 5 | Staffing and resources adequacy, nurse-doctor relationship, nurse manager ability/leadership/support of nurses, nurse participation in hospital affairs, nurse foundations for quality of care | 33 |
| Li et al. (2007), USA | 4 | Staffing and resource adequacy, nurse-doctor relationship, managerial support and leadership, opportunity for advancement, | 12 |
| Slater & McCormack (2007), Ireland | 3 | Adequate staffing and resources, nurse-doctor relationship, nurse management | 14 |
| Tervo-Heikkinen et al. (2008), Finland | 5 | Professional advancement (and support of the high managers), support of immediate superiors, staffing (and resource) adequacy, respect and relationships, standards of professional nursing | 41 |
| Gunnarsdóttir et al. (2009)  Iceland | 5 | Nurse-physician relations, unit-level support, staffing, philosophy of practice, hospital-level support | 30 |
| Van Bogaert et al. (2009), Belgium | 3 | Nurse-physician relations, nurse management at the unit level, hospital management and organizational support | 31 |
| El-Jardali et al. (2010)  Lebanon | 6 | Autonomy, control, nurse/physician relationships, organizational support, career development, participation | 27 |
| Slater et al. (2010), Ireland | 6 | Staff development and education, nursing care, adequate staff and suport, effective relationships between all staffs, nurse management, nursing involvement | 33 |
| Cho et al. (2011)  South Korea | 7 | Staffing adequacy, participation in decision-making, scheduling, education for quality care, nurse manager support, colleague support, nurse-physician relations | 39 |
| Hinno et al. (2012), Finland | 3 | Adequacy of resources, supportiveness of management, assurance of care quality via collaborative relationships | 35 |
| Kim et al. (2013)  South Korea | 6 | Participation in decision-making process, nursing process, adequate nurse staffing, education for improving quality of care, organizational support and management of hospital, physician-nurse relationship | 26 |
| Anunciada et al. (2022)  Portugal | 6 | Management support, professional development, fundamentals of nursing, nurse-physician relationship, endowments, organization of nursing care | 31 |

References:

Aiken LH, Patrician P. Measuring organizational traits of hospitals: The Revised Nursing Work Index. 2000. Nursing Research;49(3):146–153.

Anunciada S, Benito P, Gaspar F, Lucas P. Validation of psychometric properties of the Nursing Work Index-Revised scale in Portugal. 2022. International Journal of Environmental Research and Public Health;19:0-10. <https://doi.org/10.3390/ijerph19094933>

Cho S-H, Mark BA, Yun S-C, June KJ. Differences in intensive care unit work environments among and within hospitals using subscales and composite measure of the Revised Nursing Work Index. 2011. Journal of Advanced Nursing;67(12):263 –2648. <https://doi.org/10.1111/j.1365-2648.2011.05713.x>

Choi JK, Bakken S, Larson E, Du YL, Stone PW. Perceived nursing work environment of critical care nurses. 2004. Nursing Research;53(6):370– 378.

El-Jardali F, Alameddine M, Dumit N, Dimassi H, Jamal D, Maalouf S. Nurses’ work environment and intent to leave in Lebanese hospitals: Implications for policy and practice. 2010. International Journal of Nursing Studies*;*48:204–214. <https://doi.org/10.1016/j.ijnurstu2010.07.009>

Estabrooks CA, Tourangeau AE, Humphrey CK, Hesketh KL, Giovannetti P, Thomson D, Wong J, Acorn S, Clarke H, Judith S. Measuring the hospital practice environment: A Canadian context. 2002. Research in Nursing & Health;25(4):256–268. <https://doi.org/10.1002/nur.10043>

Gunnarsdóttir S, Clarke SP, Rafferty AM, Nutbeam D. Front-line management, staffing and nurse–doctor relationships as predictors of nurse and patient outcomes. A survey of Icelandic hospital nurses. 2009. International Journal of Nursing Studies;46:920–927. <https://doi.org/10.1016/j.ijnurstu.2006.11.007>

Hinno S, Partanen P, Vehviläinen-Julkunen K. The professional nursing practice environment and nurse-reported job outcomes in two European countries: A survey of nurses in Finland and the Netherlands. 2012. Scandinavian Journal of Caring Sciences;26:133–143. <http://doi.org/10.1111/j.1471-6712.2011.00920.x>

Kim C-W, Lee S-Y, Kang J-H, Park B-H, Park S-C, Park H-K, Lee K-H, Yi Y-J, Jeong B-G. Application of Revised Nursing Work Index to hospital nurses of South Korea. 2013. Asian Nursing Research;7:128–135. <https://dx.doi.org/10.1016/j.anr.2013.07.003>

Lake ET. Development of the practice environment scale of the Nursing Work Index. 2002. Research in Nursing & Health;25:176–188. <https://doi.org/10.1002/nur.10032>

Li Y, Lake E, Sales A, Sharp DN, Greiner G, Lowry E, Lui C, Mitchel P, Sochalski J. Measuring nurses practice environments with the Revised Nursing World Index: Evidence from registered health administration. 2007. Research in Nursing and Health;30:31–44. <https://doi.org/10.1002/nur.20172>

McCusker J, Dendukuri N, Cardinal L, Katofsky L, Riccardi M. Nursing work environment and quality of care: Differences between units at the same hospital. 2004. International Journal of Health Care Quality Assurance;18(7):543–551. <https://doi.org/10.1108/09526860410557561>

Slater P, McCormack B. An exploration of the factor structure of the Nursing Work Index. 2007. Worldviews on Evidence-based Nursing;4(1):30–39. <https://doi.org/10.1111/j.1741-6787.2007.00076.x>

Slater P, O’Halloran P, Connolly D, McCormack B. Testing of the factor structure of the Nursing Work Index-Revised. 2010. Worldviews on Evidence-based Nursing;7(3):123–134. <https://doi.org/10.1111/j.1741-6787.2009.00158.x>

Tervo-Heikkinen T, Partanen P, Vehviläinen-Julkunen K, Laaksonen K. Working conditions of Finnish registered nurses: A national survey. 2008. Vård I Norden;28(1):8–12. <https://doi.org/10.1177/010740830802800103>

Van Bogaert P, Clarke S, Vermeyen K, Meulemans H, Van de Heyning P. Practice environments and their associations with nurse-reported outcomes in Belgian hospitals: Development and preliminary validation of a Dutch adaptation of the Revised Nursing Work Index. 2009. International Journal of Nursing Studies;46:55–65. <https://doi.org/10.1016/j.ijnurstu.2008.07.009>
