# Supplementary material for: Organizational characteristics of nursing practice environments related to registered nurses’ professional autonomy and job satisfaction in two Finnish Magnet-aspiring hospitals: structural equation modeling study
Source: BMC Nurs. 2024 Feb 6;23:100. doi: 10.1186/s12912-024-01772-9 (PMC10845793; doi:10.1186/s12912-024-01772-9)
Supplement: Supplementary file 2 — Additional file 2. [file 12912_2024_1772_MOESM2_ESM.docx]

Supplementary file 2.

Table 1. Regression weights, unstandardized (Est.) and standardized (S.Est ).

| Variables | | | Est. | S. Est | S.E. | C.R. | p |
| --- | --- | --- | --- | --- | --- | --- | --- |
| Staffing and resource adequacy | 🡨 | Nurse management and leadership | 0.517 | 0.469 | 0.040 | 12.86 | <0.001 |
| Professional advancement | 🡨 | Nurse management and leadership | 0.631 | 0.547 | 0.043 | 14.82 | <0.001 |
| Professional advancement | 🡨 | Staffing and resource adequacy | 0.131 | 0.125 | 0.039 | 3.395 | <0.001 |
| Nursing involvement and expertise sharing | 🡨 | Nurse management and leadership | 0.412 | 0.494 | 0.032 | 12.881 | <0.001 |
| Organization’s quality standards | 🡨 | Nurse management and leadership | 0.397 | 0.475 | 0.034 | 11.538 | <0.001 |
| Collegial nurse-doctor relationships | 🡨 | Nurse management and leadership | 0.343 | 0.426 | 0.030 | 11.393 | <0.001 |
| Nursing involvement and expertise sharing | 🡨 | Professional advancement | 0.165 | 0.228 | 0.027 | 6.123 | <0.001 |
| Organization’s quality standards | 🡨 | Professional advancement | 0.117 | 0.161 | 0.029 | 4.066 | <0.001 |
| Professional autonomy | 🡨 | Nurse management and leadership | 0.122 | 0.165 | 0.031 | 3.930 | <0.001 |
| Professional autonomy | 🡨 | Nursing involvement and expertise sharing | 0.269 | 0.301 | 0.037 | 7.188 | <0.001 |
| Professional autonomy | 🡨 | Collegial nurse-doctor relationships | 0.154 | 0.166 | 0.034 | 4.579 | <0.001 |
| Professional autonomy | 🡨 | Organization’s quality standards | 0.184 | 0.206 | 0.036 | 5.116 | <0.001 |
| Job satisfaction | 🡨 | Nurse management and leadership | 1.008 | 0.314 | 0.139 | 7.238 | <0.001 |
| Job satisfaction | 🡨 | Staffing and resource adequacy | 0.458 | 0.157 | 0.103 | 4.428 | <0.001 |
| Job satisfaction | 🡨 | Organization’s quality standards | 0.449 | 0.117 | 0.156 | 2.878 | 0.004 |
| Job satisfaction | 🡨 | Professional autonomy | 1.045 | 0.242 | 0.171 | 6.093 | <0.001 |
| Professional nursing standards | 🡨 | Nurse management and leadership | 0.258 | 0.267 | 0.043 | 5.983 | <0.001 |
| Professional nursing standards | 🡨 | Professional advancement | 0.260 | 0.311 | 0.037 | 7.024 | <0.001 |

Note: Est. = regression weight; S. Est = Standardized regression weight; S.E. = standard error; C.R. = critical ratio; p = probability level.
